# Challenges and best practices for adopting and implementing digital cognitive assessments in LMICs

**DOI:** 10.1002/alz70858_097259

**Published:** 2025-12-24

**Authors:** Alexandra König, Janna Herrmann, Nicklas Linz, Johannes Tröger, Karen Blackmon, Mohamed Salama, Rashmin Gandhi, Sandra Cortés, Javiera Leniz, Irene B Meier, Vaibhav Narayan

**Affiliations:** ^1^ ki elements UG, Saarbrücken, Germany; ^2^ Memory Clinic and Research Centre, University Hospital, Nice, France; ^3^ ki:elements, SAARBRÜCKEN, Germany, Germany; ^4^ ki:elements GmbH, Saarbrücken, Germany; ^5^ Geisel School of Medicine at Dartmouth, Hanover, NH, USA; ^6^ Brain and Mind Institute, Aga Khan University, Nairobi, Kenya; ^7^ Institute of Global Health and Human Ecology at the American University in Cairo, Cairo, Cairo, Egypt; ^8^ Davos Alzheimer's Collaborative, Hyderabad, Telangana, India; ^9^ Faculty of Medicine of the Pontificia Universidad Católica de Chile, Santiago, Chile; ^10^ Departamento de Salud Pública, Escuela de Medicina, Pontificia Universidad Católica de Chile, Santiago, Chile; ^11^ Cicely Saunders Institute of Palliative Care, London, United Kingdom; ^12^ Davos Alzheimers Collaborative, Wayne, PA, USA; ^13^ Davos Alzheimer's Collaborative, Wayne, PA, USA

## Abstract

**Background:**

The rising prevalence of Alzheimer's disease (AD) in Low‐ and Middle‐Income Countries (LMICs) poses a significant public health challenge. Digital speech‐based cognitive assessments offer scalable and cost‐effective solutions for early detection and monitoring of cognitive impairment, a hallmark symptom of AD. However, their adoption and implementation face unique challenges in LMICs, including limited infrastructure, cultural and linguistic diversity, and varying levels of digital literacy. This presentation aims to outline these challenges and identify best practices for deploying digital speech‐based cognitive assessments in LMICs.

**Methods:**

As part of the Davos Alzheimer's Collaborative (DAC)'s Global Cohorts Program, ki:elements’ digital speech‐based cognitive assessment technology is being employed in four different LMICs (Kenya, Chile, Egypt, and India), involving 11.800 individuals worldwide. The technology has been adapted to all main languages spoken in the participating countries. All participants will perform a single digital speech‐based cognitive assessment which includes verbal memory and semantic fluency tasks. The resulting speech data will be analyzed jointly with demographic and clinical information to identify novel speech biomarkers from established neuropsychological tests that will enrich detection of cognitive impairment and/or underlying pathology (e.g., AD). A specific focus is placed on the effects of education level and cultural influences to ensure the technology's relevance and accuracy in diverse settings.

**Results:**

Preliminary findings show that digital speech‐based cognitive assessments are feasible and well‐accepted in diverse LMIC settings, with significant correlations to traditional cognitive tests. Key challenges include the lack of validated test norms in local languages, complexities from multilingualism and second‐language use, the impact of low education levels on task performance, and operational barriers such as limited internet connectivity. Cohort solutions have included relaxation of monolingual constraints in test administration and scoring procedures, education‐matching between cases and controls, and offline data capture. This adaptive and iterative approach, with frequent cross‐cohort communication, shows promise for enhancing the accuracy of detecting true cognitive impairment in LMICs.

**Conclusions:**

Digital speech‐based cognitive assessments offer a promising solution for addressing the growing burden of Alzheimer's disease in LMICs. However, successful implementation requires careful consideration of local infrastructure, cultural and linguistic diversity, and education levels.

